# Persistence of environmental and wastewater-associated antibiotic resistance in river water

**DOI:** 10.1007/s10661-026-15246-9

**Published:** 2026-04-10

**Authors:** Concepción Sánchez-Cid, Emilie Dehon, Carolin Schweikart, Timothy M. Vogel, Andreas Tiehm, Claudia Stange

**Affiliations:** 1 Universite Claude Bernard Lyon 1, Laboratoire d’Ecologie Microbienne, UMR CNRS 5557, UMR INRAE 1418, VetAgro Sup, 69622 Villeurbanne, France; 2 CHU de Québec-Université Laval Research Center, Endocrinology and Nephrology Axis, Québec City, Québec Canada; 3 TZW: DVGW-Technologiezentrum Wasser, Karlsruher Str. 84, 76139 Karlsruhe, Germany

**Keywords:** Antibiotic resistance, River water, Persistence, Wastewater, *Pseudomonas*

## Abstract

River water serves both as a recipient of anthropogenic antibiotic resistance (AR) via wastewater and as a reservoir of AR that can disseminate and pose risks to human and animal health. However, the fate of AR from anthropogenic and environmental sources in aquatic ecosystems remains poorly understood. In this microcosm study, we investigated the persistence of AR vectors over 10 weeks using river water from two European sites, with and without the addition of 5% (v/v) wastewater. We employed a combination of culture-based techniques, quantitative PCR, 16S rRNA gene sequencing, and genome sequencing of isolates to track both anthropogenic and environmental AR components. Our findings demonstrated the rapid decline of AR vectors associated with anthropogenic sources such as extended-spectrum beta-lactamase (ESBL)-producing *E. coli*, *bla*_TEM_, and *bla*_CMY-2_, which had a maximum half-life of 2.1 days and were below detection limits after a maximum of 20 days. In contrast, environmental markers such as ESBL-producing bacteria grown on R2A medium and *sul1* persisted over extended periods of time: their half-lives were up to 9.9 and 11.6 days, respectively, and they were detected in all samples after 10 weeks at concentrations approximately tenfold lower than at day 0. Here, we identified and characterized for the first time ESBL-producing *Pseudomonas* environmental isolates that persisted for at least 10 weeks in river water in the absence of anthropogenic input, demonstrating the pivotal role of this genus in AR maintenance in aquatic ecosystems and the need to include it in surveillance strategies to improve the accuracy of risk assessments.

**Supplementary Information:**

The online version contains supplementary material available at 10.1007/s10661-026-15246-9.

## Introduction

Antibiotic resistance (AR) has emerged as one of the most critical global health threats, with serious implications for both human and animal health (Jinks et al., 2016). Although AR is a natural phenomenon—rising as bacteria evolve mechanisms to survive naturally occurring antibiotics—human activity has significantly accelerated its development and spread in the environment (Dong et al., 2024; Larsson & Flach, 2022; Pradier & Bedhomme, 2023). Due to various sources, including domestic wastewater, hospital effluents, agricultural runoff, and sewer overflows during heavy rain events, antibiotic-resistant bacteria (ARB), antibiotic resistance genes (ARGs), and antibiotic residues are released into surface waters (Karkman et al., 2019; Łuczkiewicz et al., 2010). Wastewater treatment plants (WWTPs), although essential in reducing pollutant loads, are often unable to fully eliminate these contaminants, especially during conventional secondary treatment (Cacace et al., 2019; Jampani et al., 2024; Stange et al., 2016). In addition, rainfall may impact sewer overflow, and it has been pointed out as a driver of increased antibiotic resistance levels in the receiving environment (Amos et al., 2015). As a result, downstream aquatic ecosystems receive a complex mixture of chemical and biological contaminants, including clinically relevant ARB and ARGs, which may disseminate within microbial communities and potentially re-enter human and animal populations (Corno et al., 2019; Stange et al., 2016).

Surface waters act as both receivers of anthropogenic pollution and as hosts of diverse bacterial communities that naturally harbor AR. These environmental bacteria have adapted to various ecological conditions and often carry resistance genes unrelated to anthropogenic selective pressures (Pruden et al., 2012; Read et al., 2015). Therefore, aquatic systems contain a mixture of natural and human-derived AR, making it difficult to assess the true extent and origin of AR in these environments (Stelmaszyk et al., 2024). While antibiotics are a major driver of AR selection in aquatic ecosystems (Joannard & Sanchez-Cid, 2024; Murray et al., 2024; Sanchez-Cid et al., 2022), the persistence of ARB and ARGs in surface waters in the absence of antibiotic selection pressure has not been determined. Some studies provide evidence that plasmids carrying ARGs may be able to persist in the absence of antibiotic selection pressure (Gumpert et al., 2017; Lopatkin et al., 2017; Shi et al., 2022), while other studies suggest that these plasmids (and/or the ARGs that they carry) may be readily lost in low-impacted ecosystems (Debroas & Siguret, 2019; Finks & Martiny, 2023). Furthermore, the organic matter present in wastewater may facilitate the survival and proliferation of ARB by supporting microbial growth and enhancing opportunities for horizontal gene transfer (Jiao et al., 2017; Stange & Tiehm, 2020). Consequently, anthropogenic pollution not only introduces AR into aquatic ecosystems but may also create favorable conditions for their persistence and spread, increasing the potential risk to human and animal health. However, the long-term fate of AR in aquatic ecosystems remains poorly understood. While several microcosm studies have provided valuable insights into the survival of ARB and ARGs in water and sediment under controlled conditions (Brown et al., 2020; Calero-Cáceres & Muniesa, 2016; Kampouris et al., 2021; Mahaney & Franklin, 2022), and suggested a higher survival of ARB from human sources at colder temperatures in river water (Essert et al., 2023), important knowledge gaps regarding the differential persistence of anthropogenic versus environmental resistance, and the underlying mechanisms that govern their stability in natural settings, need to be addressed (Larsson et al., 2018; Niegowska et al., 2021).

In this study, we investigated the persistence and fate of AR in aquatic ecosystems through a large-scale (20 L) microcosm experiment. River water was collected from two contrasting central European rivers—the Rhône in France and the Alb in Germany—and incubated over a 10-week period at two temperatures (20 °C and 4 °C), with and without the addition of 5% (v/v) treated wastewater effluent. Using a combination of culture-based approaches, quantitative PCR (qPCR), 16S rRNA gene sequencing, and genome sequencing of bacterial isolates, we monitored the decay of wastewater-associated and environmental bacteria and genetic markers of resistance. We hypothesized that human-associated ARB and ARGs would not persist for long periods of time due to ecological competition, whereas environmental AR elements would show greater stability over time. This research aimed to identify key members of the community with high persistence that should be included in surveillance strategies to better assess the risks of AR dissemination.

## Materials and methods

### River water sampling and microcosm set-up

River water was sampled from two locations upstream of wastewater treatment plants: the Rhône river in Savoy (France, 45.69° N, 5.7° E) and the Alb river in Karlsruhe (Germany, 49.065° N, 8.344° E). The mean annual discharge of the River Alb is approximately 2.5 m^3^/s. The mean annual flow rate of the Rhône at the sampling site (440 m^3^/s) is significantly higher. Several WWTPs discharge effluent into both the Alb and the Rhône before the sampling point. Samples were obtained from the surface (20–50 cm depth) and 1–3 m from the riverbank. Treated wastewater was obtained from WWTPs in Chambéry (France) and Karlsruhe. The Chambéry plant is designed for 18,000 population equivalents and uses conventional treatment (mechanical treatment and secondary biological treatment with a secondary clarifier), while the Karlsruhe plant is designed for 875,000 population equivalents and uses advanced treatment (additional activated carbon filtration). For better comparability, the treated effluent was taken after secondary treatment. Microcosms (19 L) of river water were polluted with 1 L of treated wastewater. In addition, 20 L of river water microcosms without treated effluent addition were prepared in parallel and used as river water controls. Samples were incubated at room temperature (Rhône and Alb rivers) and at 4 °C (Alb river) for 10 weeks and gently shaken to emulate river currents. The temperatures were selected to represent two contrasting seasonal conditions that can occur in river ecosystems. The incubation at 20 °C reflects conditions typical of warm summer months. In contrast, the incubation at 4 °C was chosen to mimic cold winter conditions, which are relevant for assessing persistence under reduced microbial activity. Six conditions were incubated in parallel: Rhône river water at 20 °C, Rhône river water at 20 °C with 5% v/v wastewater addition, Alb river water at 20 °C, Alb river water at 20 °C with 5% v/v wastewater addition, Alb river water at 4 °C, Alb river water at 4 °C with 5% v/v wastewater addition. Duplicates were incubated in parallel for each condition. Samples were taken three times per week during the first 3 weeks and once every 2 weeks until the end of the experiment (10 weeks). Twelve samples were obtained per microcosm for a total of 144 samples (24 samples per condition). Conductivity, pH, and dissolved O_2_ concentrations were measured in all samples at each sampling time and remained constant through the experiment (Figure S1 in Supplementary Information).

### Quantification of total and extended-spectrum beta-lactamase-producing *E. coli* and river water bacteria

Total *E. coli* were quantified at each sampling time from 100 mL of water using IDEXX’s Colilert test, Quanti-Tray/2000, and Quanti-Tray Sealer according to the manufacturer’s instructions. In parallel, 100 mL of water was filtered per sample using 0.45-µm pore-size cellulose nitrate filters (Millipore). Filters were placed on CHROMagar ESBL selective media (CHROMagar) and incubated at 42 °C for 24 h before quantifying dark pink *E. coli* colonies. River water bacteria from 100 mL of Alb river microcosms incubated at room temperature and at 4 °C at every sampling time filtered using 0.45-µm pore-size filters were cultured on R2A agar medium (Merck) for 48 h at 21 °C. River water bacteria were quantified from plates without antibiotics, whereas cefotaxime 1 mg/L, ceftazidime 1 mg/L, and cefpodoxime 1 mg/L were added to quantify ESBL-producing river water bacteria, according to the method described by Stelmaszyk et al. (2024). Bacterial concentrations were normalized per volume of water to obtain cells or CFU per L.

### DNA extraction and gene quantification using qPCR

At each sampling time, 1 L of sample was filtered through a 0.22-µm pore-size filter (45 mm diameter, SO-PAK, Millipore), and DNA was extracted from the filters using the QIAmp DNA Blood Mini Kit (Qiagen) according to the manufacturer’s instructions with the following modifications as previously reported (Gourmelon et al., 2007). The filters were placed in 0.5 mL of GITC buffer and frozen at −20 °C in lysis buffer until DNA extraction. The bacterial 16S rRNA gene and ARGs commonly found in wastewater (*sul1*, *bla*_TEM_, and *bla*_CMY-2_) (Alexander et al., 2020) were analyzed by quantitative real-time PCR (qPCR) as previously described by Stelmaszyk et al. (2024). All qPCRs were performed using a Rotor-Gene cycler (Qiagen) with SsoAdvanced Universal SYBR Green Supermix (BioRad). For each assay, reactions were run in triplicate vials, and a non-template control and DNA standard with known gene copy numbers were included. The qPCR standards were generated from the serial dilutions of known quantities of linearized plasmids containing specific target genes. Baseline and threshold calculations were performed using Rotor-Gene software. Undiluted DNA, as well as 1:10 dilutions, was used as the template to ensure that negative outcomes were not the result of inhibition. No inhibitory effects were observed. All reactions had *R*^2^ values of > 0.99 and efficiencies between 90 and 105%. Furthermore, the amplification products were verified using the QIAxcel® Advanced system (Qiagen). The limit of quantification (LOQ) was ten copies per qPCR reaction for the ARGs and 100 copies per reaction for the 16S rRNA gene. Gene copies were normalized per volume of water: the LOQ was 4 × 10^3^ per L for the ARGs and 4 × 10^4^ per L for the 16S rRNA gene.

### 16S rRNA gene sequencing

The V4 hypervariable region of the 16S rRNA gene was amplified from all samples using forward (5′-TCGTCGGCAGCGTCAGATGTGTATAAGAGACAGGTGYCAGCMGCCGCGGTAA-3′) and reverse (′5-GTCTCGTGGGCTCGGAGATGTGTATAAGAGACAGGGACTACNVGGGTWTCTAAT-3′) primers with Illumina overhangs (Walters et al., 2016). DNA was amplified by PCR using the Platinum Taq DNA Polymerase (Invitrogen) and the following conditions: 94 °C for 2 min, 30 cycles of 94 °C for 30 s, 55 °C for 30 s and 72 °C for 30 s, and a final extension for 5 min at 72 °C. DNA libraries were prepared from amplified products using the Platinum Taq DNA Polymerase (Invitrogen) and the Nextera XT Index Kit V2 (Illumina) according to Illumina’s protocol for amplicon sequencing library preparation. Paired-end sequencing (2 × 250 bp) of barcoded amplicons was performed using the MiSeq System and the MiSeq Reagent Kit v2 (Illumina). Sequences were treated using the DADA2 pipeline (Callahan et al., 2016) to remove primers, trim the last ten bases of the forward reads and the last 40 bases of the reverse reads, merge forward and reverse reads, remove chimeric reads and obtain amplicon sequence variants (ASVs). ASVs were annotated taxonomically to the class level using the Ribosomal Database Project (RDP) (Cole et al., 2014). Samples that had too low sequencing depth to reach a plateau in ASV richness discovery were removed. Sequencing depths (19,298 sequences on average) are represented in Figure S2 in Supplementary material. Then, ASVs that had less than ten copies in total were removed. ASV richness was determined using the “vegan” package in R.

### Statistical analyses

XY plots were created using GraphPrism 9 to represent the abundance of the measured parameters (16S rRNA gene, ASV richness, total and ESBL *E. coli*, bacteria (including ESBL-producing bacteria) grown on R2A medium, and ARGs *sul1*, *bla*_TEM_, and *bla*_CMY-2_) over the 10 weeks of the experiment. In addition, non-metric multidimensional scaling (NMDS) analyses were run on Bray–Curtis dissimilarities calculated from ASV relative abundance from all samples at day 0 and after 1 and 10 weeks using the “vegan” package (version 2.8-0) (Oksanen et al., 2025) to identify shifts in overall community composition. PERMANOVA tests with 999 permutations were applied to determine the significance of the effects of time, temperature, river origin, and wastewater addition on bacterial community composition using the “adonis2” function of the “vegan” package in R. 16S rRNA gene abundance and ASV richness were grouped by condition regardless of time, and since they did not show a normal distribution, statistical differences between conditions were determined by Kruskal–Wallis and post hoc Dunn’s tests using GraphPrism 9. *p-*values were corrected to account for multiple comparisons (*α* < 0.05).

Finally, for each condition (Alb 20 °C river water, Alb 20 °C river water + treated wastewater, Alb 4 °C river water, Alb 4 °C river water + treated wastewater, Rhône river water, Rhône river water + treated wastewater), one-phase exponential decay equations were applied for variables related to AR (ESBL-producing *E. coli*, ESBL-producing bacteria grown in R2A medium, *sul1*, *bla*_*TEM*_, and *bla*_*CMY-2*_). Decay rates were calculated after 4 and 9 days in the Alb microcosms and after 5 and 8 in the Rhône microcosms using the following equation, where *N* is abundance at time *t*, *N°* is abundance at time 0, and *k* is the decay constant:

$$N=N^\circ\, {e}^{-kt}$$

Then, half-lives (when *N* = ½ *N*°) were calculated for each condition and time interval.

### Genome sequencing and characterization of ESBL-producing isolates from the Alb river microcosms

A selection of colonies cultivated on R2A plates with antibiotic supplementation was isolated on R2A agar plates and incubated for 24 to 48 h at 21 °C. Subsequently, taxonomic identification was conducted using MALDI-TOF MS (Bruker Daltonics) in accordance with the manufacturer’s instructions. The isolates were documented at the species level if the score value generated by the BioTyper software package (version 4.1.80, Bruker Daltonics) was between 2.0 and 3.0. Isolates were cultivated in liquid medium (R2A) at room temperature with shaking. Cultures were centrifuged and DNA was isolated from the pellet using the Fast DNA Spin Kit for Soil (MP Biomedicals) following the manufacturer’s instructions. Then, genomic libraries were prepared from each isolate according to the method described above, and isolates were sequenced on the NextSeq 1000 System using the NextSeq 1000/2000 P2 Reagent Kit 600 cycles (Illumina). Reads were quality filtered as described above, and genomes were assembled individually using MEGAHIT (Li et al., 2015). The assembled contigs were aligned against the CARD antibiotic gene database (Alcock et al., 2020) using Diamond (Buchfink et al., 2015) to identify ARGs in the isolates. Results were filtered at an amino acid identity percentage of 60%, 100 amino acid length, and an *e*-value of 10^−5^. Complete (> 98.6% completion) genomes were binned from the contigs using anvi’o (Eren et al., 2015), and genome taxonomy was determined using Centrifuge (Kim et al., 2016). Genome taxonomy, completion, and redundancy for each isolate are represented in Table S1 in Supplementary Information.

## Results

### Effect of time, temperature, river origin, and wastewater addition on river water bacterial communities in microcosms

**Fig. 1:**
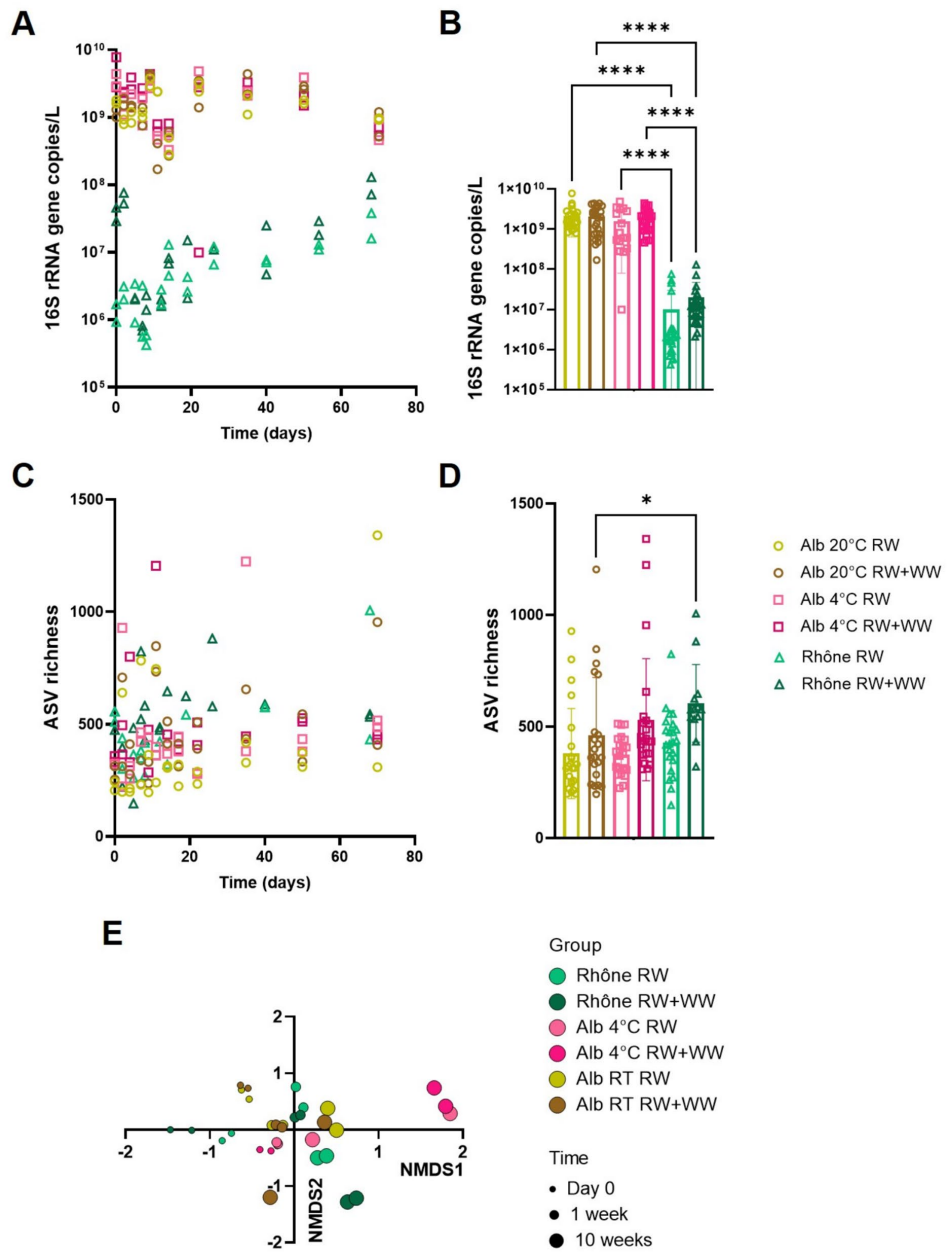
Bacterial population dynamics over time in microcosms from the Alb river at two incubation temperatures (20 °C and 4 °C) and the Rhône river at 20 °C, with or without treated wastewater addition over 10 weeks. Overall bacterial abundance estimated using a 16S rRNA gene qPCR (*N* = 136) over time (**A**) and grouped by condition (river origin, incubation temperature, and wastewater addition, **B**). Bacterial ASV richness calculated from 16S rRNA gene sequences (*N* = 126) over time (**C**) and grouped by condition (**D**). **E** Non-metric multidimensional scaling (NMDS) analysis on Bray–Curtis dissimilarities calculated from ASV relative abundance at day 0 and after 1 and 10 weeks. RW, river water. RW + WW, river water with 5% v/v wastewater addition. Dunn’s post-hoc comparisons *p-value < 0.05; **p-value < 0.01; ****p-value < 0.0001

The 16S rRNA gene was used to determine the abundance and composition of bacterial communities in river water. In the Alb river, bacterial abundance remained stable over the 10-week period (Fig. 1A), and no significant differences were found between samples incubated at room temperature and at 4 °C (Fig. 1B). Samples from the Rhône had a significantly lower bacterial abundance estimated using the 16S rRNA gene (100 to 1000-fold, *p* < 0.0001) than those from the Alb (Fig. 1B). Regarding bacterial richness, similar and stable numbers were measured in all samples (Fig. 1C). The Rhône river with wastewater addition had a significantly higher bacterial richness (*p* < 0.01) than the Alb river with wastewater addition at the same temperature (Fig. 1D). Thus, samples from the Rhône river showed a lower bacterial biomass but a similar or higher richness than samples from the Alb.

The overall bacterial community composition was evaluated in all samples at the beginning of the experiment (day 0) and after 1 and 10 weeks, in order to determine the influence of time, temperature, river origin, and the addition of wastewater (Fig. 1E). Time played the most important role in shaping bacterial community composition (PERMANOVA *R*^2^ = 0.12, *F* = 6, *p* = 0.001). River water origin (PERMANOVA *R*^2^ = 0.07, *F* = 3, *p* = 0.001) and incubation temperature (PERMANOVA *R*^2^ = 0.06, *F* = 3, *p* = 0.001) also had a limited but significant effect on bacterial communities. In the short term (1 week), incubation at 4 °C reduced community shifts in the Alb, and communities evolved differently over 10 weeks at 4 °C: Alb and Rhône bacterial communities at the same temperature (20 °C) were more similar to each other after 10 weeks than Alb communities at 4 °C and 20 °C. Finally, wastewater addition did not affect bacterial community composition (PERMANOVA *R*^2^ = 0.03, *F* = 2, *p* = 0.07), although Rhône river samples with and without wastewater addition showed a more distinct composition over the 10 weeks than samples from the Alb with and without the addition of wastewater, suggesting that wastewater pollution had a stronger impact on the lower biomass, less contaminated Rhône river than on the Alb.

### Persistence of wastewater-associated and environmental bacteria and antibiotic resistance in aquatic ecosystems

**Fig. 2:**
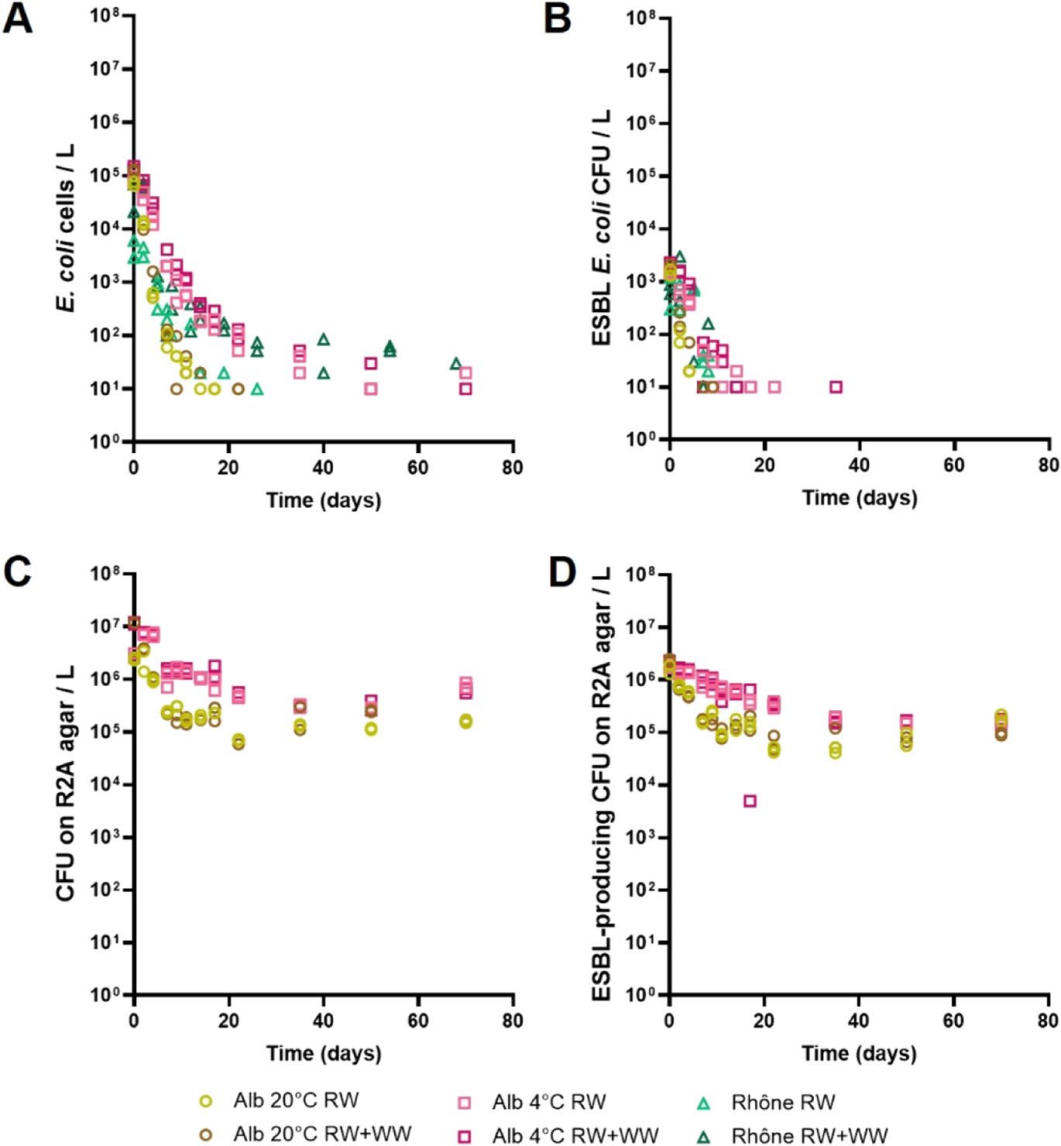
Persistence of bacterial subpopulations in microcosms from the Alb river at two incubation temperatures (20 °C and 4 °C) and the Rhône river at 20 °C, with or without treated wastewater addition over 10 weeks. **A** abundance of total *E. coli* cells quantified using the IDEXX Colilert test (*N* = 144). **B** Colony-forming units (CFU) of ESBL-producing *E. coli* grown on CHROMagar medium (*N* = 140). **C** CFU of total (*N* = 96) and **D** ESBL-producing (*N* = 96) bacteria grown on R2A agar. RW, river water. RW + WW, river water with 5% v/v wastewater addition. Values under the detection limit (1 cell/1 CFU) for graphs **A**–**D** and missing values from the Rhône for graphs **C** and **D** are not represented

**Fig. 3:**
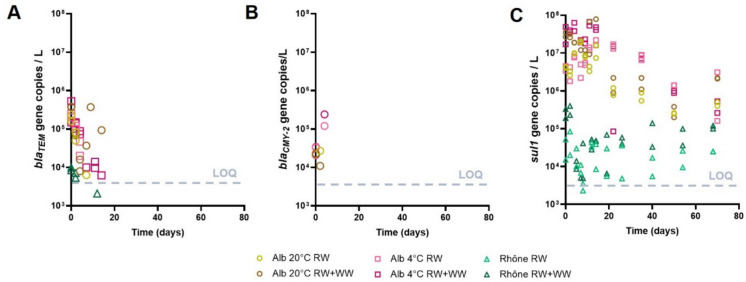
Abundance of genes coding for the antibiotic resistance genes *bla*_TEM_ (**A**), *bla*_CMY-2_ (**B**), and *sul1* (**C**), in the Alb river at two incubation temperatures (20 °C and 4 °C) and in the Rhône river at 20 °C, with or without treated wastewater and over a period of 10 weeks. RW, river water. RW + WW, river water with 5% v/v wastewater addition. LOQ, limit of quantification for qPCR analyses (4 × 10^3^ copies/L). Values under the detection limit are not represented

The abundance of bacterial subpopulations (Fig. 2) and ARGs (Fig. 3) in the two European river microcosms was monitored over 10 weeks. Overall, two distinct trends were observed over time in both rivers, independent of temperature and wastewater addition. Variables typically linked to wastewater discharge such as total *E. coli* (Fig. 2A) and ESBL-producing *E. coli* (Fig. 2B), as well as *bla*_TEM_ (Fig. 3A) and *bla*_CMY-2_ (Fig. 3B), decayed in all aquatic ecosystems. On the other hand, environmental variables commonly associated with environmental settings, such as bacteria that grow on R2A medium (Fig. 2C), including ESBL-producing bacteria (Fig. 2D), as well as the *sul1* sulfonamide resistance gene (Fig. 3C), declined slightly over time, but had a longer persistence in river water microcosms than wastewater-associated AR, and were still present at high numbers (more than 10^4^ and up to 10^7^ copies per L) at the end of the experiment.

*E. coli* was detected in samples from the Rhône and samples from the Alb incubated at 4 °C until the end of the experiment, although at levels 3–4 orders of magnitude lower than those measured at the beginning. In the Alb at room temperature, *E. coli* was no longer detected after 22 days. ESBL-producing *E. coli* decayed faster in river water and was only detected in two samples from the Alb incubated at 4 °C after day 20. Beta-lactamase genes were more abundant in the Alb than in the Rhône. In the Rhône river, *bla*_CMY-2_ was not detected in any samples, while *bla*_TEM_ was only detected in samples containing additional wastewater. Both genes were below the LOQ after the first weeks of the experiment (15–20 days for *bla*_TEM_ and by day 4 for *bla*_CMY-2_*).* Thus, all resistance markers commonly associated with wastewater showed a low persistence in river water microcosms.

First-order decay rates and half-lives were calculated at two time points during the first 2 weeks (4 and 9 days in the Alb, and 5 and 8 days in the Rhône) to quantify the initial decay of AR markers (ESBL-producing bacteria and ARGs) in river water microcosms. The role of temperature, river, and wastewater addition on this decay was also evaluated. *bla-*_CMY-2_ and *bla*_TEM_ showed the fastest decay of all variables. After 4 to 5 days, there were insufficient data above the LOQ to calculate decay rates and half-lives for *bla*_CMY-2_ (Table 1), and *bla*_TEM_ was under LOQ in several conditions after 4 to 5 days and under LOQ for all conditions studied after 8 to 9 days. In addition, ESBL-producing *E. coli*, which were initially present at slightly higher abundances in the Alb than in the Rhône (Table 1), decayed rapidly in all systems, with half-lives ranging from 0.69 to 2.1 days. This decay was faster in the Alb samples incubated at room temperature, where ESBL-producing *E. coli* was no longer detected after 9 days. All these wastewater-associated AR markers (*bla*_TEM_, *bla*_CMY-2_, ESBL-producing *E. coli*) were below the detection limit or the LOQ in all samples after 10 weeks (Table 1).

**Table 1:** First-order decay rates and half-lives of the variables measured in the river Alb and Rhône during the first 4 days (*n* = 6) and 9 days (*n* = 10)

Variable	Condition	Average concentration at day 0	*t* = 4 Alb, *t* = 5 Rhône (*n* = 6)		*t* = 9 Alb, *t* = 8 Rhône (*n* = 10)		Average concentration after 10 weeks
			Days^−1^ (*R*^2^)	Half-life	Days^−1^ (*R*^2^)	Half-life	
ESBL *E. coli*	Alb, 20 °C RW	1.5 × 10^3^ copies/L	1.00 (0.96)	0.69	N/A	N/A	< DL
	Alb, 20 °C RW + WW	1.9 × 10^3^ copies/L	0.99 (0.93)	0.70	0.58 (0.87)	1.19	< DL
	Alb, 4 °C RW	1.5 × 10^3^ copies/L	0.45 (0.94)	1.54	0.50 (0.95)	1.39	< DL
	Alb, 4 °C RW + WW	1.9 × 10^3^ copies/L	0.33 (0.94)	2.10	0.57 (0.82)	1.22	< DL
	Rhône, RW	7.5 × 10^2^ copies/L	−0.46 (0.03)	*	0.40 (0.61)	1.73	< DL
	Rhône, RW + WW	7.4 × 10^2^ copies/L	0.80 (0.68)	0.87	0.55 (0.62)	1.26	< DL
ESBL-producing bacteria in R2A medium	Alb, 20 °C RW	1.6 × 10^6^ CFU/L	0.24 (0.87)	2.89	0.23 (0.85)	3.01	1.5 × 10^5^
	Alb, 20 °C RW + WW	2.4 × 10^6^ CFU/L	0.4 (0.92)	1.73	0.30 (0.93)	2.31	1.0 × 10^5^
	Alb, 4 °C RW	1.9 × 10^6^ CFU/L	0.07 (0.6)	9.90	0.09 (0.76)	7.70	1.4 × 10^5^
	Alb, 4 °C RW + WW	2.3 × 10^6^ CFU/L	0.11 (0.88)	6.30	0.10 (0.90)	6.93	1.6 × 10^5^
*sul1* gene copies	Alb, 20 °C RW	4.2 × 10^6^ copies/L	−0.21 (0.47)	*	−0.14 (0.48)	*	4.6 × 10^5^
	Alb, 20 °C RW + WW	3.1 × 10^7^ copies/L	0.12 (0.70)	5.78	0.12 (0.85)	5.775	2.0 × 10^6^
	Alb, 4 °C RW	4.0 × 10^6^ copies/L	−0.18 (0.32)	*	−0.03 (0.03)	*	1.6 × 10^6^
	Alb, 4 °C RW + WW	3.3 × 10^7^ copies/L	−0.13 (0.28)	*	0.06 (0.22)	11.55	4.0 × 10^5^
	Rhône, Rhône, RW	3.4 × 10^4^ copies/L	0.11 (0.10)	6.3	0.28 (0.60)	2.48	2.5 × 10^4^
	Rhône, RW + WW	2.6 × 10^5^ copies/L	0.66 (0.78)	1.05	0.48 (0.71)	1.44	1.1 × 10^5^
*bla*_TEM_ gene copies	Alb, 20 °C RW	1.9 × 10^5^ copies/L	0.88 (0.98)	0.78	N/A	N/A	< LOQ
	Alb, 20 °C RW + WW	3.8 × 10^5^ copies/L	N/A	N/A	N/A	N/A	< LOQ
	Alb, 4 °C RW	2.3 × 10^5^ copies/L	0.50 (0.89)	1.38	N/A	N/A	< LOQ
	Alb, 4 °C RW + WW	3.4 × 10^5^ copies/L	0.31 (0.66)	2.23	N/A	N/A	< LOQ
	Rhône, RW	< LOQ	N/A	N/A	N/A	N/A	< LOQ
	Rhône, RW + WW	9.15 × 10^3^ copies/L	N/A	N/A	N/A	N/A	< LOQ
*bla*_*CMY-2*_ gene copies	Alb, 20 °C RW	< DL	N/A	N/A	N/A	N/A	< LOQ
	Alb, 20 °C RW + WW	2.3 × 10^4^ copies/L	N/A	N/A	N/A	N/A	< LOQ
	Alb, 4 °C RW	2.7 × 10^4^ copies/L	N/A	N/A	N/A	N/A	< LOQ
	Alb, 4 °C RW + WW	2.78 × 10^4^ copies/L	N/A	N/A	N/A	N/A	< LOQ
	Rhône, RW	< LOQ	N/A	N/A	N/A	N/A	< LOQ
	Rhône, RW + WW	< LOQ	N/A	N/A	N/A	N/A	< LOQ

*R*^2^ values for each exponential decay curve are provided in parentheses. *N/A* not assessed due to values under the detection limit (< DL) or limit of quantification (LOQ). *RW* river water. *RW + WW* river water with 5% v/v treated wastewater. Negative values represent parameters that increased over time. *Half-life could not be determined since decay rates were negative (value increasing over time). ESBL-producing bacteria on R2A agar were not quantified in the Rhône

In addition, the number of ESBL-producing bacteria grown on R2A agar (only determined in the Alb river) also decreased over time, especially in samples polluted with treated wastewater and incubated at 20 °C. However, their decay rates were lower than those of ESBL-producing *E. coli*, with half-lives ranging between 1.73 and 9.9 days for ESBL-producing bacteria grown on R2A medium (Table 1). By the end of the experiment, their abundance in the Alb river microcosms was ~ 10 times lower than at the beginning. Finally, *sul1* exhibited high background levels in all rivers, although it was two orders of magnitude higher in the Alb than in the Rhône. The addition of treated wastewater increased *sul1* abundance by a factor of 10 in all systems (Table 1). This gene showed a high persistence in the Alb, with half-lives reaching up to 11.55 days, although final concentrations were ten times lower than at day 0. In the Rhône river microcosms, the decay rates for *sul1* were higher in the anthropogenically polluted samples during the first week. At day 70, both microcosm experiments—with and without treated wastewater—had similar *sul1* levels as at day 0 in river water without the addition of treated wastewater. Overall, *sul1* was associated with both background resistance and anthropogenic input, demonstrating high persistence in our model aquatic ecosystems over time. Parameters associated with wastewater decayed quickly in our microcosms, whereas parameters associated with environmental communities and genes persisted over time. Incubation at 4 °C slowed the decrease in the abundance of total and ESBL-producing *E. coli* and bacteria grown on R2A medium in the Alb river during the first week compared to incubation at room temperature, although this effect was not observed for antibiotic resistance gene abundance.

### Long-term persisting antibiotic-resistant bacteria isolated from river water contain extended-spectrum beta-lactamases

Eighteen ESBL-producing bacteria cultured on R2A medium were isolated from the Alb river microcosms at the last three sampling times, and their genomes were sequenced to determine the resistome of the persistent resistant members of the river water community (Table 2). Eighty-nine percent of the isolates were annotated as *Pseudomonas*, whereas one isolate at day 35 was identified as *Janthinobacterium lividum*, another isolate at day 35 was identified as *Paenibacillus amylolyticus*, and one isolate at day 50 was identified as *Flavobacterium hydatis.* Ten *Pseudomonas* species were identified: *P. koreensis*, *P. frederiksbergensis*, *P. brennerii*, *P. chlororaphis*, *P. fluorescens*, *P. amylolyticus*, *P. antactica*, *P. proteolytica*, *P. jessenii*, and *P. corrugate.* Among these, only *P. koreensis* was isolated from samples at all three sampling times. All isolates contained RND efflux pumps involved in beta-lactam resistance, with the exception of *Flavobacterium*. All *Pseudomonas* strains contained the *mexAB-oprM* efflux pump, and 72% contained *parRS*. Overall, ESBL genes were present in all isolates. *Janthinobacterium* had *THIN-B* and *rm3*, *Flavobacterium* had *JOHN-1* and *OXA* beta-lactamases, and *Pseudomonas* isolates had *PDC* beta-lactamases (12% of the isolates), *CRP* beta-lactamases (63% of the isolates), or both (25% of the isolates). All *Pseudomonas* isolated from microcosms after 5–10 weeks harbored genetic mechanisms involved in the resistance to carbapenems, penams, cephalosporins, monobactams, penems, and cephamycins, whereas *Janthinobacterium* and *Flavobacterium* lacked genes involved in monobactam, penem, and cephamycin resistance (Table 2). Thus, bacterial isolates producing ESBLs and carrying genes involved in the resistance to six beta-lactams were detected in the Alb river after 35, 50, and 70 incubation days.

**Table 2:**
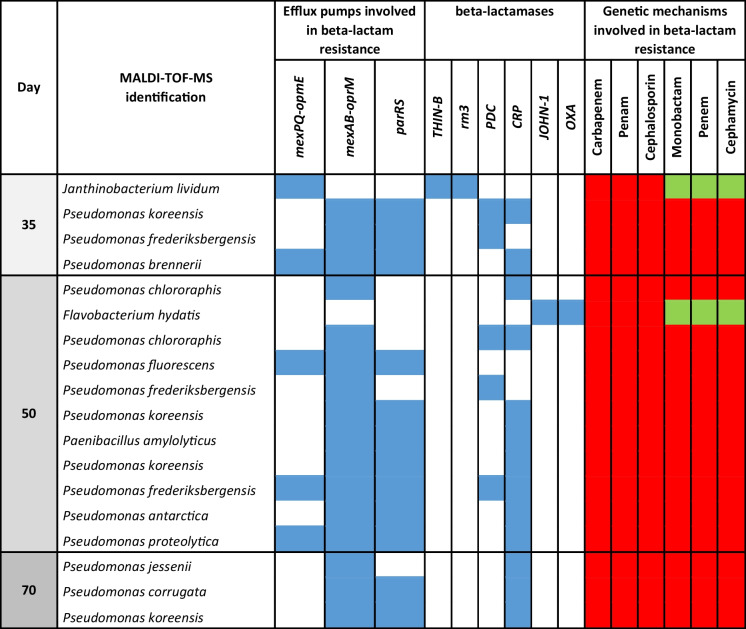
Taxonomy and presence (blue) or absence (white) of beta-lactamases and efflux pumps involved in beta-lactam resistance and the presence (red) or absence (green) of the associated genetic resistance mechanism for various beta-lactams (carbapenems, penams, cephalosporins, monobactams, penems, and cephamycins) in the ESBL oligotrophic isolates obtained from Alb river samples after 35, 50, and 70 incubation days

## Discussion

The goal of this study was to determine the persistence of environmental AR markers associated with wastewater and aquatic ecosystems in the absence of continuous anthropogenic input from wastewater treatment plants, as that may increase the risk of AR dissemination from environmental settings to human microbiomes over the long term, even in low-impacted ecosystems. We evaluated the impact of various factors, including two river waters in Europe with different levels of biomass and anthropogenic input, two incubation temperatures, and the addition of wastewater, on this persistence in large-scale microcosms. Although all of these factors contributed to shaping the bacterial communities in river water and altering the abundance of AR markers (ARB and ARGs), our research showed a generalized trend across conditions. Wastewater-associated AR markers (ESBL-producing *E. coli*, *bla*_TEM_, and *bla*_CMY-2_) decreased rapidly in our model aquatic ecosystems in the absence of anthropogenic pressure, whereas environmental markers of AR commonly found in aquatic ecosystems (ESBL-producing bacteria grown on R2A medium and *sul1*) showed a higher persistence.

Although some of these ARB/ARGs in the microcosms were introduced via wastewater, the highest proportions of them (including both wastewater-associated and environmental markers) were already present in the river water controls (which are sampled upstream of the WWTP used in this study but downstream of other WWTPs). Thus, while certain genes and bacteria may be introduced via wastewater, their long-term persistence in the absence of continuous anthropogenic input depends on their compatibility with native microbial communities and ecological niches. River water endogenous bacteria growing on R2A medium are likely to be less dependent on nutrients than fecal bacteria (Governal et al., 1991). In addition, other compounds released with wastewater may impose a stress on aquatic communities that could favor the long-term ecological success of human-associated bacteria*,* as illustrated in river water biofilms exposed to copper (Bagra et al., 2023). Another possible explanation for the lack of long-term success of wastewater-associated AR markers is the reduced probability of niche colonization in diverse endogenous communities. However, recent findings suggest that this ecological phenomenon frequently found in more stationary environments such as soil plays a negligible role in river water ecosystems (Klümper et al., 2024). The success of ARGs in river water ecosystems may also be linked to their ability to integrate stable genetic frameworks and their compatibility with diverse hosts in aquatic ecosystems. While beta-lactamases carried by human-associated bacteria seem to be rapidly lost along with their hosts in river water microcosms, *sul1* is known to be an environmental gene often linked to class 1 integrons and mobile genetic elements (Abramova et al., 2023; Wei et al., 2018). Its long-term survival in environmental settings is attributed to horizontal gene transfer (Muziasari et al., 2014). The combination of the high persistence and the high potential for horizontal gene transfer, as well as the frequent detection of *sul1* in different environments, highlights the need to include this gene in AR surveillance.

This is the first study to identify and characterize environmental isolates that showed a phenotypic resistance to last-generation beta-lactamases and that persisted for at least 10 weeks in river water microcosms in the absence of anthropogenic pressure. These bacteria were primarily found to be associated with the genus *Pseudomonas,* which is widely distributed in environmental ecosystems (Camiade et al., 2020) and includes opportunistic pathogens such as *P.* *fluorescens* (Picot et al., 2001; Scales et al., 2014). Bacteria of the genus *Pseudomonas* are known to possess various AR mechanisms, such as beta-lactamases, efflux pumps, and aminoglycoside-modifying enzymes, that contribute to multidrug resistance in clinical and environmental settings (Camiade et al., 2020; Yang et al., 2024). Frequently described resistance mechanisms in *Pseudomonas* include RND efflux pump genes, such as those identified in all of our isolates (Lister et al., 2009), as well as *Pseudomonas*-derived cephalosporinase (PDC) genes, which confer high levels of phenotypic resistance (Rodríguez-Martínez et al., 2009), identified in one third of the isolates. Furthermore, genes from the CRP family (class A beta-lactamases) conferring resistance to cephalosporins (Gudeta et al., 2016) were detected in almost all of the *Pseudomonas* isolates. Although these beta-lactam resistance mechanisms are chromosomally encoded, *Pseudomonas* have, as Gram-negative γ-*Proteobacteria*, the capacity to share AR plasmids with *Enterobacteriaceae* (Laroche-Ajzenberg et al., 2015). While *Pseudomonas* may exhibit relatively low rates of horizontal gene transfer to *Enterobacteriaceae* (Camiade et al., 2020), their ecological stability and ubiquity in aquatic ecosystems may increase the probability of acquisition and dissemination of ARGs by *Pseudomonas* in aquatic environments. In addition, *Pseudomonas* plays a major role in shaping the resistome in response to anthropogenic stress in aquatic ecosystems (Dehon et al., 2025). Their persistence in the absence of anthropogenic pressure indicates an intrinsic capacity to maintain resistance, even in ecosystems with minimal human impact. In light of these findings, we encourage both the inclusion of the genus *Pseudomonas* in the surveillance of environmental AR reservoirs, beyond species associated with infections in clinics, and future research needed to address the frequency and mechanisms of AR dissemination from these environmental strains of *Pseudomonas* to human pathogens.

These findings demonstrate the underestimated importance of AR markers such as ESBL-producing *Pseudomonas* and the *sul1* gene on the long-term persistence of AR in aquatic ecosystems. To avoid interference from continuous anthropogenic discharge from WWTPs, this study used a microcosm approach, which has inherent limitations compared to dynamic river water ecosystems. Some biotic and abiotic factors such as exposure to natural sunlight and predation have been shown to influence bacterial survival in river water (Dean & Mitchell, 2022). For example, antibiotic-resistant *Pseudomonas* and *E. coli* have been found in amoeba in surface waters (Delumeau et al., 2023), and they can survive phagocytosis by these eukaryotic predators and be protected from environmental stress. In addition, consistent with previous findings (Chern et al., 2022), *E. coli* and bacteria grown in R2A medium (including ESBL-producing members) exhibited a slower decay at colder temperatures. Therefore, seasonality is expected to play a role in the long-term persistence of AR markers in aquatic ecosystems. Further research should address the long-term persistence of AR in situ and quantify the contribution of the multiple physiochemical factors that drive this persistence in natural ecosystems, to create quantitative models of dissemination risk to bacteria in the human microbiome.

## Conclusion

Our study showed that AR parameters associated with wastewater from anthropogenic sources, such as ESBL-producing bacteria and the beta-lactamase genes *bla*_TEM_ and *bla*_CMY-2_, are not able to persist in model aquatic ecosystems over long periods of time (at least 10 weeks) in the absence of continuous anthropogenic input. In contrast, antibiotic-resistant bacteria such as ESBL-producing *Pseudomonas* and environmental ARGs that are commonly found in the environmental resistome were able to persist. To understand the dynamics of AR from a One Health perspective, the recognition of the central role of surveillance is essential. A considerable proportion of surveillance programs for aquatic environments is focused on clinically relevant antibiotic-resistant bacteria. For instance, the monitoring of ESBL-producing *E. coli* has been identified as a common practice (Anjum et al., 2021; Berendes et al., 2020; World Health Organization, 2021). In contrast, AR from environmental sources that are regularly found in the environmental resistome and may persist in these aquatic ecosystems are rarely considered. The biased use of anthropogenic parameters may lead to an underestimation of the environmental dimension of the risk of AR as part of a One Health approach. In particular, the spread in low-to-moderately polluted aquatic ecosystems, which are common in Europe, could be underestimated. Based on the results of our study, we recommend that future environmental surveillance strategies include AR markers of environmental relevance that are able to colonize and persist in the environment even at low levels of anthropogenic pollution. The detection of persisting bacteria such as ESBL-producing *Pseudomonas* that play a critical role in the composition of the aquatic resistome and might disseminate ARGs to human pathogens can provide important information to improve risk assessment and mitigation strategies.

## Supplementary Information

Below is the link to the electronic supplementary material.

Supplementary file1 (DOCX 116 KB)

## Data Availability

The datasets generated and analyzed during the current study are available at the DDBJ repository, BioProject PRJDB20170, DRA accession DRA020141. All codes used in this study is available at: https://github.com/concscid/Sanchez-Cid-et-al-2025-persistenceAR.
