# Supplementary material for: Persistence of environmental and wastewater-associated antibiotic resistance in river water
Source: Environ Monit Assess. 2026 Apr 10;198(5):429. doi: 10.1007/s10661-026-15246-9 (PMC13065532; doi:10.1007/s10661-026-15246-9)
Supplement: Supplementary file 1 — Supplementary file1 (DOCX 116 KB) [file 10661_2026_15246_MOESM1_ESM.docx]

**Supplementary Information**

**Environmental Monitoring and Assessment**

**Persistence of environmental and wastewater-associated antibiotic resistance in river water**

Concepcion Sanchez-Cid^1^, Emilie Dehon^1,2^, Carolin Schweikart^3^, Timothy M. Vogel^1^, Andreas Tiehm^3^, Claudia Stange^3^

^1^Universite Claude Bernard Lyon 1, Laboratoire d'Ecologie Microbienne, UMR CNRS 5557, UMR INRAE 1418, VetAgro Sup, 69622 Villeurbanne, France

^2^CHU de Québec-Université Laval Research Center, Endocrinology and Nephrology Axis, Québec City, Québec, Canada

^3^TZW: DVGW-Technologiezentrum Wasser, Karlsruher Str. 84, 76139 Karlsruhe, Germany

*Corresponding author: [sanchezcidtorres@gmail.com](mailto:sanchezcidtorres@gmail.com)

**
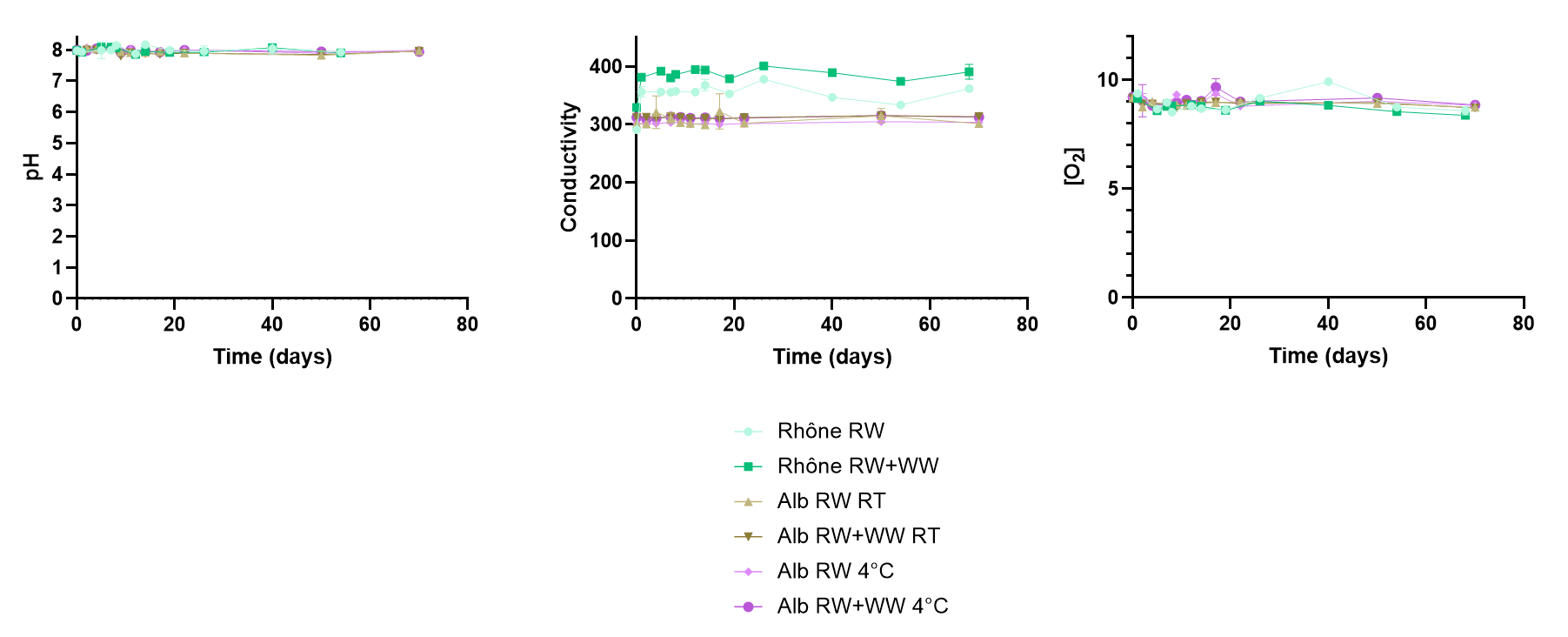
**

**Figure S1.** pH, conductivity and dissolved oxygen concentration in the Alb and the Rhône rivers over the 10-week incubation.

**Figure S2.** Sequencing depths by condition after removing samples that did not meet a plateau of ASV richness discovery due to too low sequencing depth. Alb 20°C RW *n*=24; Alb 20°C RW+WW *n*=24; Alb 4°C RW *n*=22; Alb 4°C RW+WW *n*=20; Rhône RW *n*=17; Rhône RW+WW *n*=18).

| **Day** | **MALDI-TOF-MS**  **identification** | **Genome taxonomy** | **Completion (%)** | **Redundancy (%)** |
| --- | --- | --- | --- | --- |
|  |  |  |  |  |
| **35** | *Janthinobacterium lividum* | *Janthinobacterium* | 98.6 | 0 |
|  | *Pseudomonas koreensis* | *Pseudomonas* | 98.6 | 0 |
|  | *Pseudomonas frederiksbergensis* | *Pseudomonas* | 100 | 4.2 |
|  | *Pseudomonas brennerii* | *Pseudomonas* | 100 | 0 |
| **50** | *Pseudomonas chlororaphis* | *Pseudomonas* | 98.6 | 4.2 |
|  | *Flavobacterium hydatis* | *Flavobacterium* | 100 | 4.2 |
|  | *Pseudomonas chlororaphis* | *Pseudomonas* | 98.6 | 0 |
|  | *Pseudomonas fluorescens* | *Pseudomonas* | 98.6 | 4.2 |
|  | *Pseudomonas frederiksbergensis* | *Pseudomonas* | 100 | 0 |
|  | *Pseudomonas koreensis* | *Pseudomonas* | 100 | 1.4 |
|  | *Paenibacillus amylolyticus* | *Pseudomonas* | 100 | 1.4 |
|  | *Pseudomonas koreensis* | *Pseudomonas* | 100 | 2.8 |
|  | *Pseudomonas frederiksbergensis* | *Pseudomonas* | 100 | 0. |
|  | *Pseudomonas antarctica* | *Pseudomonas* | 100 | 1.4 |
|  | *Pseudomonas proteolytica* | *Pseudomonas* | 100 | 0 |
| **70** | *Pseudomonas jessenii* | *Pseudomonas* | 98.6 | 0 |
|  | *Pseudomonas corrugata* | *Pseudomonas* | 98.6 | 2.8 |
|  | *Pseudomonas koreensis* | *Pseudomonas* | 100 | 1.4 |

**Table S1.** Taxonomy, completion and redundancy of the genomes sequenced from isolates from Alb river water microcosms grown on R2A medium.
